# Transparent and Fine Film Stencils with Functional Coating for Advanced Surface Mount Technology

**DOI:** 10.3390/mi16090969

**Published:** 2025-08-22

**Authors:** Byoung-Hoon Kang, Wonsoon Park, Kyungjun Park, Hunjoong Lee, Junjong Yoo, Namsun Park, Chulyong Jung

**Affiliations:** Global Technology Research, Samsung Electronics Co., Ltd., Suwon 16677, Republic of Korea

**Keywords:** surface mount technology, film stencil, clear polyimide, diamond-like carbon coating, miniaturization

## Abstract

Technological advancements for the miniaturization of electronic components highlight a critical role of thin and durable stencils in advanced surface mount technology. Here, we report a transparent and fine film stencil consisting of a clear polyimide film and a functional diamond-like carbon coating layer for the fine-pitch surface mount technology process. High-quality and burr-free apertures in the thin film result from the thermally stable laser-cut process using a repetitive and low-power irradiation of nanosecond pulse laser, enhancing the printing accuracy of solder paste with fewer solder joint defects. The carbon coating layer with an electrostatic discharge composition facilitates smooth and robust surfaces and sidewalls of the apertures for the high solder paste release and high mechanical durability of the fine film stencil. The low-cost and easy fabrication of the fine film stencil accelerates the potential industrial replacement of the conventional metal stencils at a reduced thickness and further open a new opportunity for the mass production of the fine-pitch surface mount technology process.

## 1. Introduction

Metal stencils in surface mount technology (SMT) provide accurate and uniform solder paste printing for the assembly of electronic components onto printed circuit boards (PCBs) [1,2]. Recently, the increasing demand for ultra-miniaturized and high-density packaging poses significant challenges for conventional metal stencils due to the technical trade-off between the thickness of metal stencils and the mechanical durability [3]. The downscaling of electronic components demands a corresponding decrease in the solder paste thickness, requiring the thinner metal stencil; however, it suffers from the mechanical degradation and inconsistent paste release [4]. In addition, the conventional metal stencils fabricated by laser cutting, or chemical etching, exhibit high wall roughness in the apertures, which leads to poor solder release [5]. Thus, new stencils with advanced materials are required in order to achieve sufficient durability and high printing quality at an even reduced thickness [6,7].

Unlike conventional metal stencils, electroforming stencils have emerged as a promising solution for thinner stencil thickness and fine-pitch SMT processes [8]. Furthermore, the uniform and layered deposition of nickel onto a patterned substrate enables smooth aperture sidewalls and burr-free clean surfaces, which significantly enhance the paste release property and printing accuracy [9,10,11]. However, the bottom-up fabrication methods remain impractical for mass production in SMT processes due to the high fabrication cost, lengthy processing time, and increased process complexity [12]. In particular, structural limitations, such as the non-uniform current distribution, limited scalability of electroforming bath, and reduced mechanical stability, make it challenging to ensure a uniform thickness and precise pattern formation in a large area [13].

State-of-the-art materials offer promising inspiration as an alternative of the metallic stencils for fine-pitch applications [7,14,15]. Film stencils utilizing low-cost and mass-producible polymers, such as polyethylene terephthalate (PET) and polyimide (PI), have been actively attempted in order to improve the solder paste release by using its smooth aperture surface. However, the flexible film stencils face critical technical hurdles, including thermal degradation from the laser cut, limited long-term reliability, and severe warpage in a large area, which still pose significant challenges for mass production [14]. Recently, a polyimide stencil masks with a lateral resolution of 42.4 μm have improved the reproducibility and turnaround time with high aspect ratios at a thickness of below 100 μm; however, the wafer-scale polymer stencil still hinders the long-term reliability and their applicability in the fine-pitch and large-area production for the SMT process [15].

**Figure 1:**
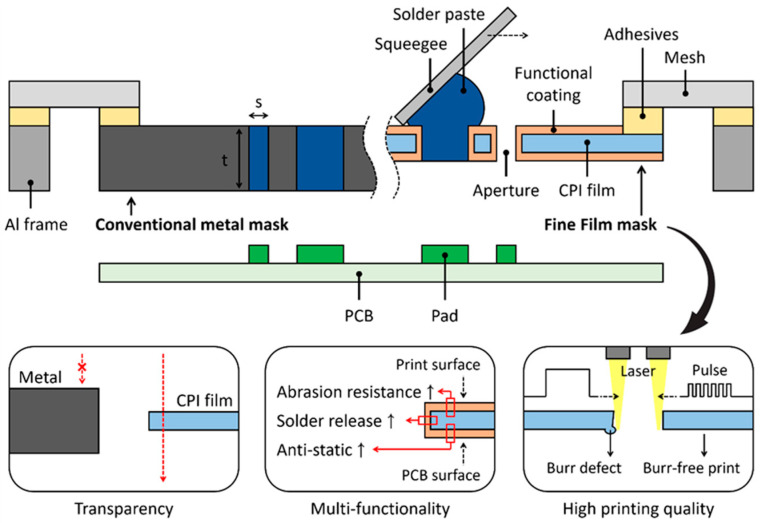
Schematic illustration of conventional metal stencil and transparent and fine film stencil for advanced SMT process. Schematic illustration of the conventional metal stencil and the fine film stencil. The thickness (t) of stencil is required to be reduced in accordance with miniaturization of aperture size (s). The fine film stencil comprises CPI film, functional coating, adhesives, and mesh-linked Al frame and allows transparency, high abrasion resistance, high solder release efficiency, anti-static functionality, and high printing quality.

Here, we report a transparent, robust, and fine film stencil suitable for the mass production of fine-pitch SMT (Figure 1). The fine film mask consists of a clear polyimide (CPI) film, laser-cut apertures, a functional coating, and a mesh-linked aluminum (Al) frame. The thermostable and transparent CPI film allows not only high-quality and burr-free printing of the solder paste at a reduced thickness, but also real-time monitoring for precise alignment and defect inspection. The uniform functional coatings, such as diamond-like carbon (DLC) and electrostatic discharge (ESD) coating, provides superior solder paste release, high durability, and enhanced printing conformity by covering both sides and the sidewalls of the apertures. This fine film stencil demonstrates the reliable transfer efficiency of the solder paste during repetitive SMT cycle tests and is expected to be suitable for substituting the metal stencil due to the time-saving and cost-effective fabrication process.

## 2. Materials and Methods

### 2.1. Fabrication of Fine Film Stencil

**Figure 2:**
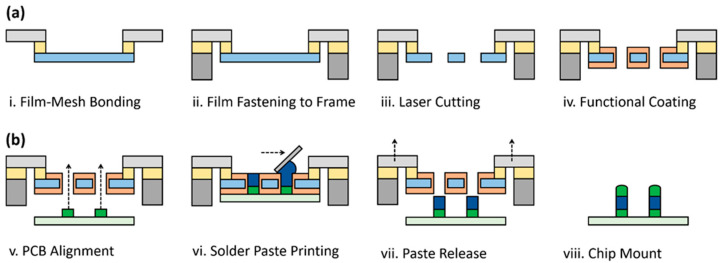
Schematic illustration of (**a**) fabrication procedures and (**b**) application methods of the fine film stencil for the SMT process. The fine film stencil is simply made by sequential protocols of film-mesh bonding, film fastening to Al frame, laser cutting, and functional coating.

The fabrication process of the fine film stencil involves film-mesh bonding, film fastening to Al frame, laser cutting, and functional coating (Figure 2a). The roll-type CPI film was first prepared into sheets according to the physical dimension of 640 mm × 540 mm for the SMT stencil. The film thickness of 80 μm was specifically selected to meet the requirements of the mobile device, which can be reduced to 50 μm depending to the target application. The CPI film is commonly used due to its superior characteristics of lower internal stress, higher thermal stability, and lower surface friction coefficient, compared to the conventional polymer materials [16]. The CPI sheet adhered to the polyester mesh through the use of an adhesive, which provides uniform tension to maintain flatness of flexible stencil and distribute mechanical stress during repeated uses. The chloroprene (CR)-based adhesive is commonly used for adhering screen-print fabric to the metal stencil with a solid content of 30% and a viscosity of 3700 cps. The film–mesh composite was strongly stretched under high tensile force in biaxial directions and integrated into the 10 mm-thick Al frame using the adhesive. The mesh tension for the vertical displacement at a center under tension is 1.5 mm. The through-holes on the film surface were formed as the apertures by using ultraviolet (UV) nanosecond-pulse laser with repetitive and low-power irradiation to minimize thermal deformation of the polymer film, with 20 repetitions with a power of 0.8 W, a speed of 50 mm/s, a beam size of 20 μm, and a pulse width of 20 ns. An ESD-DLC coating was finally applied to both surfaces of the stencil and sidewalls of the apertures by using sputtering deposition. The DLC coating was deposited using a graphite pulse arc sputtering system with methane (CH_4_) as a reactive gas at pressures ranging from 10^−4^ Pa to 10^−1^ Pa, with a hardness of 7000 HV, a heat resistance to 300 degrees Celsius, and a thickness of 200 nm. Note that the ESD protection was added to prevent static electricity of electronic components by an addition of doping elements like silicon or nitrogen during the DLC coating process [17]. All fabrication procedures of the fine film stencils were automated for the mass production.

### 2.2. Application Methods of Fine Film Stencil

The fully packaged fine film stencil demonstrates solder paste printing for reflow soldering of surface-mount chip components on a circuit board (Figure 2b). The fine film stencil was precisely aligned and tightly contacted with the PCB for matching between the apertures and solder pads. Note that the conventional SMT process requires additional fiducial marks in both the PCB and the stencil [18]; however, the transparent fine film stencil helps optical alignment and real-time monitoring of defects without any additional patterns, enhancing the deposition efficiency of solder paste. The molten solder paste was uniformly distributed to fill the apertures by squeegeeing with consistent pressure and speed across the stencil. The fine film stencil was then lifted after the solder paste printing to allow clean separation of the solder paste from the apertures. The SMT printing process was performed with a printing pressure of 6 kgf, a printing speed of 40 mm/s, a separation speed of 1 mm/s, and a separation distance of 4 mm with automatic cleaning performed after every two boards. The electronic components were finally positioned onto the printed solder pastes by using a pick-and-place machine and thermally reflowed in a reflow soldering oven for reliable electrical connections between the pads and the chips.

## 3. Results and Discussions

### 3.1. Experimental Verifications for Laser Cutting Quality

**Figure 3:**
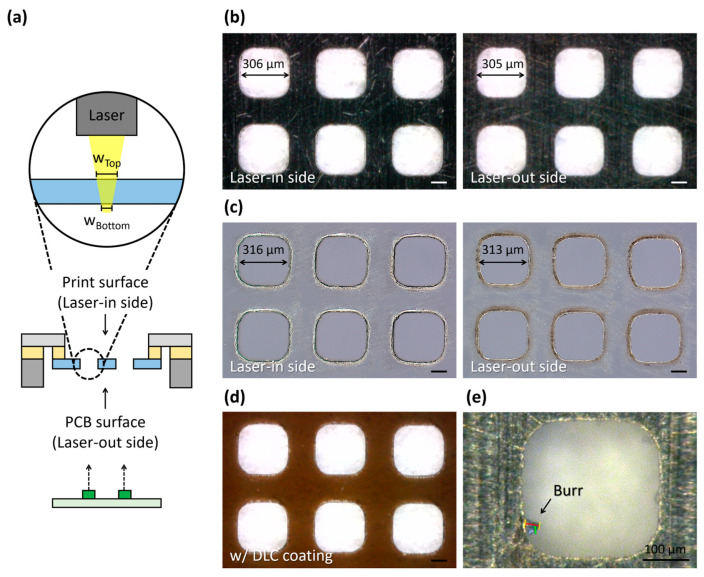
Comparison of laser cutting quality including morphological analysis, taper accuracy, and burr presence between conventional metal stencil and fine film stencil. (**a**) Schematic diagram of experimental condition for measuring the laser cutting quality: laser-in top side for print surface of solder paste, and laser-out bottom side for PCB surface. Microscopic images of (**b**) the metal stencil and (**c**) the fine film stencil on the laser-in and the laser-out sides. (**d**) Microscopic image of the fine film stencil after DLC coating. (**e**) Microscopic image of the burr presence in the metal stencil, with 32.88 μm of burr size; the scale bar corresponds to 100 μm.

The fine film stencil demands high laser cutting quality comparable to the conventional metal stencil for the fine-pitch mass production. The laser cutting quality of the fine film stencil was evaluated by using a morphological, kerf taper, and burr formation analysis (Figure 3a). A laser beam is irradiated onto a squeegee-contacting print surface opposite to a PCB-contacting surface, resulting in a difference in the kerf width at the top and bottom of a laser-cut due to a taper effect: a wider top kerf width (w_top_) for the laser-in side and a narrow bottom kerf width (w_bottom_) for the laser-out side. The power and speed of the laser beam was experimentally determined for high laser cutting quality; a higher laser power and a lower laser speed reduce the kerf taper angles, resulting in an improvement in the taper characteristics and kerf uniformity. For the laser cutting process for a square pattern with a width of 300 μm and rounded corners, the conventional metal stencil exhibits a uniform and well-defined morphology despite the presence of surface scratches (Figure 3b). The measured w_top_ and w_bottom_ are 306 μm and 305 μm, respectively, with a slight kerf width difference of 1 μm. The fine film stencil demonstrates the reliable morphology of laser-cut apertures with an increased kerf width difference of 3 μm, which meets the quality standard of a difference under 10 μm (Figure 3c). Note that the taper angle of the sidewalls causing non-uniform solder paste release can be reduced by using laser trepanning methods to enable an adjustment of the beam displacement and inclination, or the spatial beam shaping method to use the Bessel beam [19,20].

The suboptimal shapes and the heat-affected zones at the edges of the laser-out side have little impact on the printing quality with no vertical step deformation, which can be improved by the DLC coating and the optimization of the laser processing conditions. In particular, the DLC layers reduce the squeegee-driven wear by covering the surface scratches and the heat-affected zones on both surfaces of the fine film stencil (Figure 3d). Note that the burring and heat-affected zones in laser-cut patterns can be improved to reduce thermal effects by using ultrashort-pulsed lasers including the femtosecond laser instead of the nanosecond laser; it may increase the overall manufacturing cost and unit price in mass production [21,22].

### 3.2. Experimental Verifications for Minimizing Burr Formation

The burr formation frequency is measured by counting the edge burrs in a sample of 4000 pads. The edge burrs are observed as unwanted raised edges or polymer residues along the aperture sidewalls, which are normally formed during the laser cutting process. The size and shape of burrs vary irregularly depending on the laser parameters and material properties. The conventional metal stencil shows edge burrs up to 40 μm in size with the formation rate of 0.1–1% (Figure 3e), whereas the fine film stencil exhibits a significantly low burr formation rate of approximately 0.025% with small sizes, with typically a height of about 5 μm and a width of about 10 μm. The burr formation rate of the fine film stencil is 4–40 times better than the conventional metal stencil. Note that the low-power laser irradiation repeated 20 times on the same area contributes to reducing the burr formation frequency by minimizing the thermal damage to the CPI film [23]. As a result, the fine film stencil achieves high-quality and burr-free apertures suitable for reliable solder paste printing at a reduced thickness.

### 3.3. Experimental Verifications for Solder Paste Release

**Figure 4:**
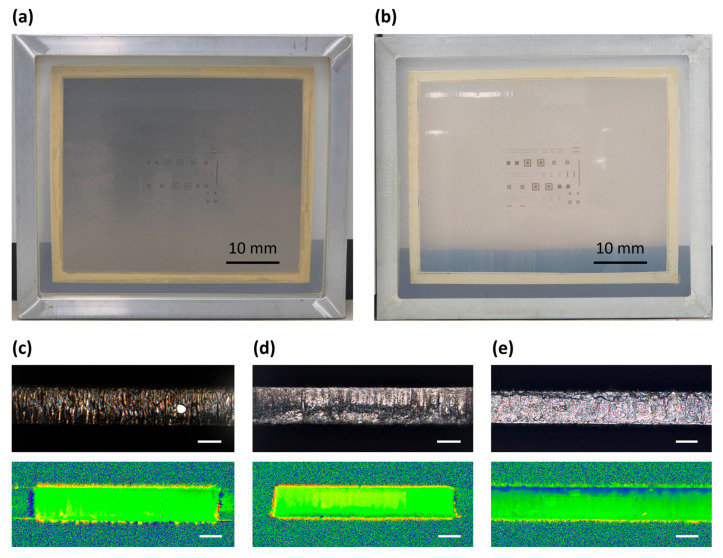
Comparison of surface roughness between conventional metal and fine film stencil for sidewall of apertures. Captured photographs of (**a**) the fully packaged metal stencil and (**b**) the fully packaged fine film stencil. (**c**) Microscopic image and height map of surface on the sidewall of aperture for the metal stencil. Microscopic images and height maps of fine film stencils (**d**) before and (**e**) after the DLC coating. The fine film stencil with the DLC coating provides superior surface roughness, which improves solder paste release; the scale bar corresponds to 50 μm.

The solder paste release from the small apertures of the stencil plays a crucial rule for uniform and reliable paste deposition. The smooth sidewalls of the apertures with low friction and anti-static surface properties can reduce paste residues after printing and improve the transfer efficiency of the solder paste. The solder paste release is evaluated between the conventional metal stencil and the fine film stencil by measuring the sidewall roughness of the aperture with a sample number of 5 (Figure 4a,b). The sidewall roughness is defined using an arithmetic mean height (Sa) value as the average surface roughness in a three-dimensional space, which is considered acceptable according to the quality standard when below 0.6 μm. The measured Sa value of the conventional metal stencil is 0.834 μm, which can degrade the printing quality due to its rough surface (Figure 4c). For the CPI film, the measured Sa value of the fine film stencils before and after the DLC coating are 0.628 μm and 0.379 μm, respectively (Figure 4d,e). Note that the other film materials commonly show an increase in the sidewall roughness due to thermal degradation during the laser cut, i.e., 2.03 μm for polyether ether ketone film; however, the CPI film with the high thermal stability provides superior surface roughness compared to the metal stencils.

The experimental results also indicate that the DLC coating on the sidewall of apertures exhibits a substantial improvement of 2.6 times and 1.7 times in the sidewall roughness compared to the metal stencils and the CPI film, respectively, allowing for a high printing quality due to the improved solder paste release. The thickness of the DLC layer on the aperture sidewalls is commonly determined as 5–15% of the surface deposition thickness [24], which is sufficient for improving the solder release while maintaining the printed solder volume. In addition, the ESD composition is applied as the ESD coating with a surface resistivity of about 4.5 × 10^3^ ohms per square, which protects sensitive electronic components from damage and ensures reliable and high-quality assemblies.

### 3.4. Experimental Verifications for Spatial Alignment of Apertures

**Figure 5:**
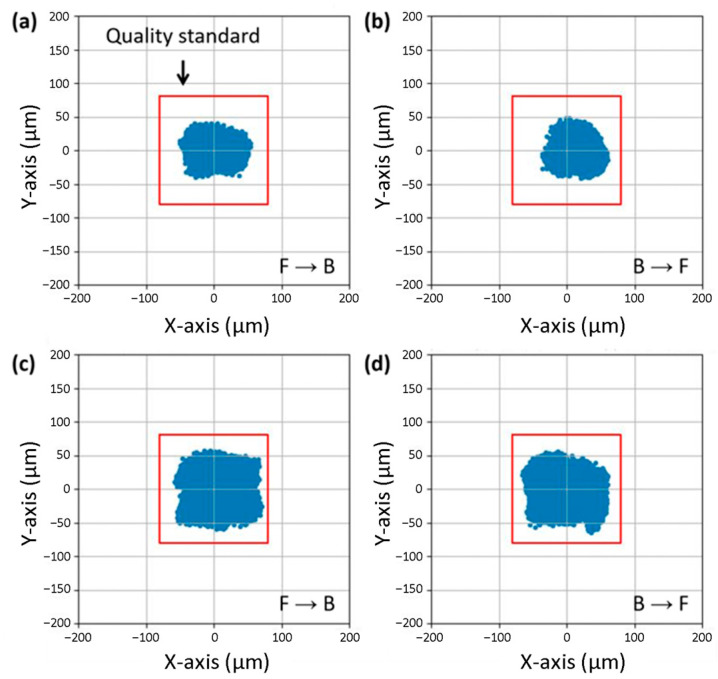
Comparison of spatial alignment for uniformity in a large area. Scattering plot of two-dimensional alignment quality in (**a**) front-to-back and (**b**) back-to-front print direction for conventional metal stencil. Scattering plot of two-dimensional alignment quality in (**c**) front-to-back and (**d**) back-to-front print direction for fine film stencil. The red box represents quality standard of ±80 μm for SMT process.

The spatial alignment between the laser-cut apertures of the stencil and the patterns of the test board was evaluated for both the conventional metal stencil and fine film stencil. The test board includes various sizes (100–500 μm), geometries, and patterns of apertures of 197,460 with a large board size of 150 mm × 150 mm. A scattering plot of two-dimensional alignment quality was used to evaluate the positional consistency of solder paste printing with two squeegee directions of front-to-back and back-to-front. For the conventional metal stencil, the standard deviation (σ) values of the spatial alignment along the X-axis and Y-axis were 15.68 μm and 15.39 μm in the front-to-back direction, respectively (Figure 5a). The σ values along the X-axis and Y-axis were 17.63 μm and 11.62 μm in the back-to-front direction, respectively (Figure 5b). In comparison, the fine film stencil exhibited the σ values of 23.43 μm for the X-axis and 16.76 μm for the Y-axis in the front-to-back direction (Figure 5c). The fine film stencil also showed the σ values of 23.02 μm for the X-axis and 21.69 μm for the Y-axis in the back-to-front direction (Figure 5d). The fine film stencil represents the anisotropy of solder paste printing with a higher X-axis variability compared to the Y-axis due to the intrinsic mechanical compliance and stretchability of the CPI film under the squeegee-driven shear force. In other words, the film undergoes a slight lateral deformation in the squeegee direction, resulting in an increase in X-axis deviation. The Y-axis perpendicular to the squeegee movement is less affected by the dynamic shear. Despite the observed differences in alignment, the positional deviation for both the metal and fine film stencil remained within the acceptable manufacturing tolerances for the standard SMT components, ±80 μm for quality standard. Note that the positional deviation of up to 100 μm does not induce the misconnection of the solder joint within the allowable tolerance range for general-purpose chip components in standard SMT applications. The experimental results show that the fine film stencil provides sufficient positional accuracy for the large-area SMT process.

### 3.5. Experimental Verifications for Printing Quality in Repeated SMT Process

**Figure 6:**
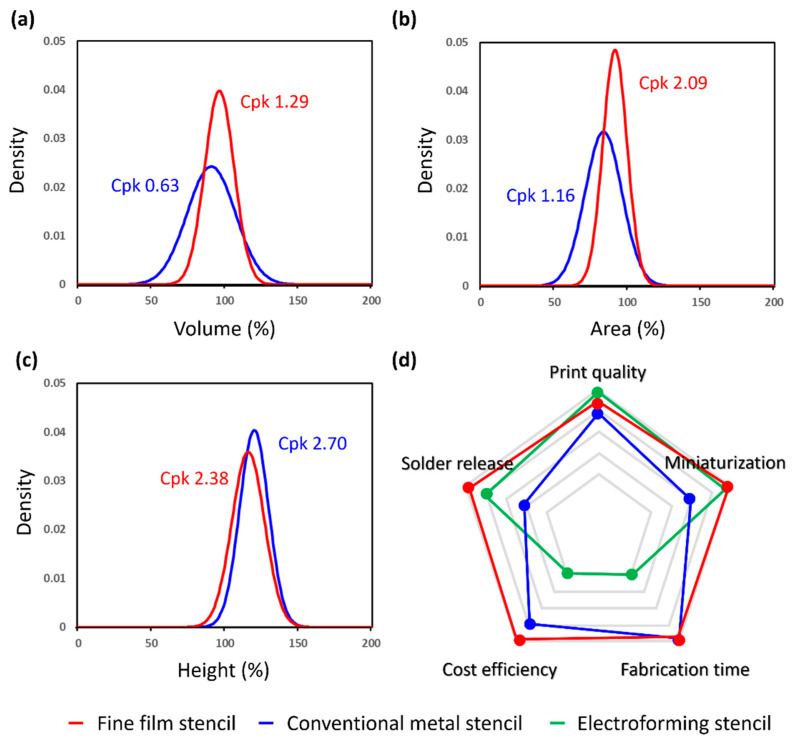
Comparison of print quality between fine film stencil and conventional metal stencil. Distribution of solder paste percentage for (**a**) volume, (**b**) area, and (**c**) height after repeated SMT process using fine film stencil and conventional metal stencil. (**d**) Pentagonal radar chart for the comparison of fine film stencil and the other conventional metal stencils. The fine film stencil satisfies the SMT requirement of solder paste release, durability, printing quality, cost efficiency, and fabrication efficiency.

The consistent printing quality of the solder paste for the high-quality SMT soldering was finally performed by using the fully-packaged metal and fine film stencils for a target solder joint thickness of 80 μm. The squeegee-driven printing speed and pressure force are 25 mm/s and 8 kgf, respectively. The fine film stencil is separated from the PCB by a distance of 3 mm at a speed of 0.5 mm/s after the solder paste printing. The process capability index (Cpk) is used to evaluate the transfer efficiency of solder paste printing by comparing the key print quality parameters of volume, area, and height [25]. The Cpk values were calculated based on the measured distribution of the printed solder paste volumes, areas, and heights relative to the specified tolerance limits with a total of 30 samples. For the solder paste volume, the fine film stencil showed a Cpk of 1.29 with a mean value of 94.15% and standard deviation of 8.81, while the metal stencil had a lower Cpk of 0.63 with a mean of 90.84 and standard deviation of 16.44 (Figure 6a). The fine film stencil delivered a higher and more consistent paste volume than the metal stencil and achieved the near-capability threshold of 1.33, which represents reliable stability in the solder paste release. For the area, the film stencil outperformed with a Cpk of 2.09, a mean of 91.55%, and a standard deviation of 8.23, compared to the metal stencil, which had 1.16 for the Cpk, 83.71 for the mean, and 12.6 for the standard deviation (Figure 6b). The high Cpk value of the fine film stencil reflected the more distinct aperture boundary and precise alignment accuracy due to the optical transparency, thus enhancing the lateral resolution and reducing edge defects. For the height, both stencils showed a high process capability over 1.33 as the acceptable threshold, and a Cpk of 2.38 for the fine film stencil and a Cpk of 2.70 for the metal stencil (Figure 6c). As a result, the conventional metal stencil exhibits a slightly higher Cpk value in height distribution, whereas the fine film stencil allows superior Cpk values in both the volume and area with a sufficiently high Cpk value of height. Thus, the experimental results demonstrate overall superior print quality, which is particularly advantageous at the reduced stencil thicknesses required for the fine-pitch applications.

The experimental results for the printing quality evaluation also demonstrated that the fine film stencil exhibits a superior process capability with high printing quality and high consistency, and even outperforms the conventional metal stencils for the amount of excessive and insufficient solders at the target solder thickness of 80 μm, with 238 parts per million (ppm) and 43,518 ppm of excess and insufficient solders for the metal stencil, but 0 and 223 ppm of excess and insufficient solders for the fine film stencil in the total solder amount of 197,460. In addition, the fine film stencil showed a comparatively long-term durability of at least 4000 times for squeezing and printing without any visual defect and physical deformation. The fine film stencil also maintained the surface properties of anti-static function and abrasion resistance after multiple cleaning cycles using both water-based and ethanol-based cleaning solutions.

### 3.6. Comparison Between Conventional Metal Stencils and Fine Film Stencil

A pentagonal radar chart shows a comparison among the conventional stainless stencil, the electroforming nickel stencil, and the fine film stencil for print quality, solder paste release, miniaturization, cost efficiency, and fabrication efficiency (Figure 6d). The print quality is assessed by the Cpk values using a three-dimensional measurement of the solder paste volume, which indicates the repeatability and consistency. The fine film stencil showed a significantly better value of Cpk, indicating improved print uniformity. The solder release is evaluated by comparing the sidewall roughness for the transfer efficiency of the solder paste. The fine film stencil with the DLC coating represented a high enhancement in the release properties due to the reduced surface energy and smoother sidewalls. The miniaturization capability is determined based on the stencil thickness and aperture size ratio. The fine film stencil allows for a finer resolution due to their lower thickness under 85 μm without a structural compromise, whereas metal stencils face limitations below 100 μm due to the mechanical instability and poor paste release in high-aspect-ratio apertures. As a result, the fine film stencil exhibits a superior print quality and solder release compared to the stainless stencil at a reduced thickness of 85 μm, while offering a faster and more cost-effective fabrication than the electroforming nickel stencil, thereby improving the process efficiency. In other words, the fine film stencil satisfies the SMT requirement of printing performance, cost efficiency, and fabrication efficiency at the same time. Furthermore, the fine film stencils using a thinner CPI film under 50 μm is expected to deliver comparable durability and printing quality, potentially accelerating the miniaturization and high-density packaging in the SMT processes. The fine film stencil, with further technical developments, has many potential strengths for the advanced SMT: its large-area thickness uniformity, improvement in coordinate distortion, and low shifts in physical tension for superior long-term reliability comparable to the metal stencil (about 100,000 runs per a stencil).

## 4. Conclusions

In summary, this work has successfully demonstrated a mass production of the fine-pitch SMT process using a transparent, robust, and fine film stencil for the high transfer efficiency of the solder paste, high printing quality, and high durability. The fine film stencil consisting of a transparent CPI film contributes to high-quality and burr-free laser-cut apertures with reliable performances of the morphology and kerf taper angle. The functional protective layers of a DLC and ESD coating significantly enhance the sidewall roughness by 2.6 times better than the metal stencil and improve the solder paste release with a high durability during repetitive SMT processes. The fully packaged fine film stencil demonstrates the high process capability of SMT by about 2 times and a superior defective rate by about 200 times better than the fully packaged metal stencil. This miniaturized and cost-efficient platform can contribute to the technical improvement in the fine-pitch printing of the solder paste and the high-density packaging of small electronic components. For full industrial viability, the fine film stencil requires further validations including long-term reliability under harsh thermal, mechanical, and corrosive conditions, dimensional stability during repeated squeegee operations, and the material diversification of cost-effective polymer, and further holds potential as a paradigm shift for diverse assembly applications requiring the reduced thickness.

## Data Availability

The data will be made available upon request.
